# Spectrum of Urological Surgeries in a Nigerian Specialist Hospital: A Three-Year Review

**DOI:** 10.7759/cureus.93519

**Published:** 2025-09-29

**Authors:** Ifeanyichukwu E Ihedoro, Paul E Ngwu, Chibueze P Ohiarah, Enyinnanya V Onyemachi

**Affiliations:** 1 Urology, Eleos Specialist Hospital, Umuahia, NGA; 2 Urology, North Cumbria Integrated Care NHS Foundation Trust, Carlisle, GBR; 3 Urology, Federal Medical Centre Umuahia, Umuahia, NGA; 4 Surgery, Federal Medical Centre Umuahia, Umuahia, NGA

**Keywords:** minimally invasive, nigeria, procedure, surgery, urology

## Abstract

Introduction: Urology deals with the management of medical conditions affecting the male genitourinary tract and the female urinary tract. A wide range of procedures may be performed in the management of these diseases. The aim of this study is to analyze the indications for urological procedures performed in a urology specialist hospital, with a view to providing vital data that can be used as a policy framework for healthcare development in the country.

Methods: The records of patients who underwent urological procedures between January 2022 and December 2024 were retrieved from the hospital's Medical Records Department. Vital information retrieved from case files was analyzed using Statistical Product and Service Solutions (SPSS, version 29; IBM SPSS Statistics for Windows, Armonk, NY).

Results: A total of 1,253 urological procedures were done between the years under review. This comprises 1,086 males (86.7%) and 167 females (13.3%). The mean age of participants was 61.3±15.7 years. The three most common indications for surgery were benign prostatic hyperplasia (BPH) (29.9%), renal/ureteric calculi (22%), and suspected prostate cancer (9.7%). The most common procedures performed were transurethral resection of the prostate/bipolar enucleation of the prostate (27.1%), laser lithotripsy (15.3%), and prostate biopsy (9.7%). Additionally, 65.5% of the procedures were minimally invasive, 71.8% of them were performed under regional anaesthesia, 67.1% of the patients required inpatient stay, and 94.1% were elective cases.

Conclusion: Most of the surgeries were performed endoscopically, mainly for BPH and urolithiasis.

## Introduction

Urology as a specialty deals with the diagnosis, treatment, and management of surgical and medical conditions affecting the male genitourinary tract and the female urinary tract [1]. There is a wide array of procedures in the management of urological conditions, ranging from diagnostic, therapeutic, to reconstructive interventions. These conditions include benign prostatic hyperplasia (BPH), urolithiasis, malignancies, and congenital anomalies, among others [2]. Tertiary hospitals, serving as referral centers for complex cases, require advanced surgical expertise, appropriate technology, and multidisciplinary care to address the varied patient needs [3]. A grasp of the range of urological procedures, including information on their demographic patterns, types, clinical indications, anaesthesia modalities, care delivery settings, invasiveness, and urgency, is important in improving healthcare delivery, patient outcomes, and guiding health policy development as regards urology practice. In resource-poor settings such as Nigeria, tertiary centers face challenges such as limited access to endoscopic surgeries, minimally invasive technologies, and delayed patient presentations, which shape treatment patterns compared to developed nations with more advanced diagnostic and therapeutic capacities [4]. The aim of this study is to analyze the indications for urological procedures performed in Eleos Specialist Hospital Umuahia, Nigeria, and compare this with the patterns in tertiary centers in Nigeria, with a view to providing vital data that can be used as a policy framework for healthcare development in the country and beyond.

## Materials and methods

This study is a retrospective study of all urological surgeries performed over a three-year period at Eleos Specialist Hospital, Umuahia, between January 2022 and December 2024. Ethical approval was obtained from the Health Research Ethics Committee of the hospital (ELEOS/REC/2025/007). The facility is a 30-bedded urology specialist hospital that provides tertiary-level urology care in Abia State, Southeastern Nigeria. It runs a busy outpatient clinic that holds twice a week and consults 40 patients on average per day. The hospital has two modular theatres and is equipped to carry out a variety of urology procedures, including open and minimally invasive procedures. Patients booked for elective cases are assessed in the clinic by the surgeon and admitted to the ward a day before surgery. They were reviewed by the anaesthetist a day before their surgery. Patients who presented as an emergency were optimised and reviewed by the surgeon and anaesthetist before surgery. Informed consent was obtained from all the patients before surgery. Data were obtained from the booking register and theatre records and transferred into a proforma. The information retrieved included biodata (age, sex), type of procedure, indication of procedure, anaesthesia, type of admission, and surgical approach. The data were analyzed using Statistical Product and Service Solutions (SPSS, version 29; IBM SPSS Statistics for Windows, Armonk, NY). Continuous variables were reported as means with corresponding standard deviations, while categorical variables were summarized as frequencies and proportions.

## Results

A total of 1,253 surgeries were carried out during the study period. One thousand and eighty-six of the patients were males (86.7%), while 167 (13.3%) were females. The mean age of participants was 61.3±15.7 years, as shown in Table 1. The three most common indications for surgery were BPH (29.9%), renal/ureteric calculi (22%), and suspected prostate cancer (9.7%), as shown in Table 2. The most common procedures performed were transurethral resection of the prostate/bipolar enucleation of the prostate (27.1%), laser lithotripsy (15.3%), and prostate biopsy (9.7%), as shown in Table 3. Minimally invasive procedures (endoscopic and laparoscopic surgeries) accounted for 821 (65.5%) of the surgeries done. Regional anaesthesia was the most common form of anaesthesia used during surgeries. It accounted for 899 (71.8%), followed by local anaesthesia in 292 (23.3%) and general anaesthesia in 63 (4.9%) of the procedures performed. Eight hundred and forty-one patients (67.1%) had inpatient admission, while 32.9% of the surgeries were performed as day case surgeries. One thousand, one hundred and seventy-nine (94.1%) of the procedures were elective cases, while only 74 (5.9%) were emergency procedures.

**Table 1:** Age and Sex Distribution of the Study Participants

Variables	Frequency, n= 1253	Percent (%)
Age group (years)			
<30	55	4.4
30-39	80	6.4
40-49	107	8.5
50-59	168	13.4
60-69	465	37.1
70-79	292	23.3
≥80	86	6.9
Mean ± SD	61.3 ± 15.7	
Sex		
Male	1086	86.7
Female	167	13.3

**Table 2:** Diagnosis of the Study Participants

Diagnosis	Frequency, n= 1253	Percent (%)
Urethral stricture	63	5.0
Urinary retention	27	2.2
Renal/ ureteric calculi	276	22.0
Testicular torsion	19	1.5
Varicocele	24	1.9
Suspected Prostate cancer	122	9.7
BPH	375	29.9
Prostate Cancer/testicular cancer	85	6.8
Bladder neck stenosis	15	1.2
Bladder stone	26	2.1
Hematuria/ Suspected bladder Cancer	66	5.3
Bladder cancer	48	3.8
Hernia	29	2.3
Hydrocele	33	2.6
Prostate cancer	11	0.9
Pelviureteric junction obstruction	4	0.3
Renal Cancer	11	0.9
Others	19	1.5

**Table 3:** Procedures Done on the Study Participants DVIU: Direct vision internal urethrotomy; TURP: Transurethral resection of the prostate; TURBT: Transurethral resection of bladder tumor

Procedures	Frequency, n= 1253	Percent (%)
DVIU	59	4.7
Urethroplasty	4	0.3
Suprapubic cystostomy	27	2.2
DJ Stenting & removal	47	3.8
Laser lithotripsy	193	15.3
Percutaneous nephrolithotomy	36	2.9
Scrotal exploration	19	1.5
Varicocelectomy	24	1.9
Prostate biopsy	122	9.7
TURP/Bipolar enucleation of the prostate	339	27.1
Simple open prostatectomy	36	2.9
Orchidectomy	85	6.8
Bladder Neck Incision/ wedge resection	15	1.2
Cystolitholapaxy/ cystolithotripsy	26	2.1
Cystoscopy	66	5.3
TURBT	48	3.8
Herniorrhaphy	29	2.3
Hydrocelectomy	33	2.6
Radical prostatectomy (open and laparoscopic)	11	0.9
Pyeloplasty (Laparoscopic & open)	4	0.3
Nephrectomy & nephroureterctomy	11	0.9
Others	19	1.5

## Discussion

The mean age of patients in this study was 61.3±15.7 years. This is similar to findings of related studies done in the country [5,6]. In hospitals where pediatric urological surgeries are performed, they often involve children under 12 years, with a median age of 3.2 years [7,8]. Most of the surgeries were performed in males, with a male-female ratio of 12.5:1. The gender distribution of urological procedures in different centers varies, with one study reporting a male-female ratio of up to 65:1 [9]. This shows that the majority of the patients undergoing urological procedures are males, mainly as a result of the high prevalence of prostate disease.

Prostate-related disease was the commonest indication for a surgical procedure in our study, with BPH being the most prevalent. This is similar to the findings noted among studies done in other parts of Nigeria [10,11]. Idowu et al. in Oyo, Nigeria, reported that benign prostate enlargement was the most common indication for inpatient surgeries, whereas suspected prostate cancer was the leading indication for outpatient procedures [10]. This is an indicator of the relatively high burden of prostate disease in the region. The other indications for surgery observed are similar to those reported in other studies in the country [5,9,10].

In our study, the most common procedures performed were transurethral resection of the prostate/bipolar enucleation of the prostate (27.1%), laser lithotripsy (15.3%), and prostate biopsy (9.7%). This is in contrast to the study conducted by Idowu et al. [10], where the most common in-patient urological procedure was open simple prostatectomy (14.1%) for benign prostatic hypertrophy, while the most common outpatient urological procedure was prostatic needle biopsy (32.74%) for suspected prostate cancer [10]. In another study by Tela et al. in a Nigerian Tertiary hospital, the commonest procedure was cystoscopy, followed by orchidectomy for prostate cancer [12]. A similar study from a Nigerian tertiary hospital revealed that digital-guided prostate biopsy (37.6%) was the most common procedure, followed by suprapubic cystostomy (12.8%) and open prostatectomy (7.2%) [5]. Findings from our study reveal the gradual shift towards endoscopic procedures for treatment of BPH and urolithiasis as the mainstay treatment modality for management of BPH and upper urinary tract stones in Nigeria. Hospitals in the developed world prioritize endoscopic and robotic procedures due to improved access to the needed technology. A UK study reported that an increasing proportion of procedures done in their country were endoscopic, and there is a rapid geographical adoption of robotic surgery, with the majority of providers already performing laparoscopic interventions before adopting robotic surgery for prostate and kidney cancers [13,14].

In our study, most patients had regional anaesthesia, followed by local and general anaesthesia. This is similar to findings in Nigerian tertiary hospitals [5,6]. Regional anaesthesia is considered to be cheaper and more appropriate for elderly patients with comorbidities [5,6]. General anaesthesia is used for major surgeries such as nephrectomy and radical prostatectomy, but its limitations include the availability of anaesthesiologists and anaesthetic drugs. Local anaesthesia is common for day case procedures, including outpatient cystoscopy and minor biopsies [6].

In-patient procedures accounted for the majority of surgeries done in our hospital, as patients were admitted for a couple of days for observation before discharge. However, in many Nigerian hospitals, day cases predominate, making up to 70% of the total urological procedures in some centers [5,6]. In several Nigerian hospitals, procedures such as prostate needle biopsy [5,10] and cystoscopy [12] were the most common urological surgeries and were performed as day case procedures. Hospitals in developed countries prioritize day case surgery, with the majority of procedures performed on an outpatient basis, made possible by adequate postoperative care systems and minimally invasive techniques [3].

Most of the surgeries done in our hospital were endoscopic. This is in contrast to findings in several Nigerian hospitals where open procedures dominate, accounting for up to 90% of urological interventions in some centers [5,7,9,10]. This clearly highlights the growing availability of skills and equipment for endoscopic urological procedures in Nigeria. Endourology comes with the advantages of less functional impairment of target organs, shorter inpatient stays, and early return to activities, ultimately leading to improved patient outcomes [15].

Majority of urological surgeries in our study were elective procedures. This is similar to studies done in the country, where most of the urological surgeries were elective procedures [10,12]. The commonest emergency surgeries include suprapubic cystostomy, scrotal exploration, percutaneous nephrostomy, and debridement for Fournier’s gangrene, in several studies in the region [11].

## Conclusions

Prostate-related disease was the commonest indication for a surgical procedure in our study, while endoscopic surgery for BPH and urolithiasis was the predominant treatment modality. This highlights the burden of prostate diseases in our region and the attendant need for more research and funding.

There is a gradual shift towards endoscopic techniques as the mainstay of treatment for certain urological conditions in our country. However, there is still a need for improved training and provision of necessary equipment in other tertiary hospitals to meet the growing demand.
